# 483. Pharmacokinetics of Nirmatrelvir and Ritonavir in COVID-19 Patients with Hemodialysis

**DOI:** 10.1093/ofid/ofad500.553

**Published:** 2023-11-27

**Authors:** Xin Li, Huajian Ying, Feng Sun, Yaxin Fan, Yi Li, Xiaofen Liu, Mengting Chen, Beining Guo, Jun Xue, Jiming Zhang, Jing Zhang

**Affiliations:** Institute of Antibiotics, Huashan Hospital, Fudan University, Shanghai, China, Shanghai, Shanghai, China; Department of Nephrology, Huashan Hospital, Fudan University, Shanghai, 200040, China, Shanghai, Shanghai, China; Department of Infectious Diseases, Huashan Hospital, Fudan University, Shanghai, 200040, China, Shanghai, Shanghai, China; Institute of Antibiotics, Huashan Hospital, Fudan University, Shanghai, 200040, China; Key Laboratory of Clinical Pharmacology of Antibiotics, National Health and Family Planning Commission, Shanghai 200040, China, Shanghai, Shanghai, China; Institute of Antibiotics, Huashan Hospital, Fudan University, Shanghai, 200040, China; Key Laboratory of Clinical Pharmacology of Antibiotics, National Health and Family Planning Commission, Shanghai 200040, China, Shanghai, Shanghai, China; Institute of Antibiotics, Huashan Hospital, Fudan University, Shanghai, 200040, China; Key Laboratory of Clinical Pharmacology of Antibiotics, National Health and Family Planning Commission, Shanghai 200040, China, Shanghai, Shanghai, China; Institute of Antibiotics, Huashan Hospital, Fudan University, Shanghai, 200040, China; Key Laboratory of Clinical Pharmacology of Antibiotics, National Health and Family Planning Commission, Shanghai 200040, China, Shanghai, Shanghai, China; Institute of Antibiotics, Huashan Hospital, Fudan University, Shanghai, 200040, China; Key Laboratory of Clinical Pharmacology of Antibiotics, National Health and Family Planning Commission, Shanghai 200040, China, Shanghai, Shanghai, China; Department of Nephrology, Huashan Hospital, Fudan University, Shanghai, 200040, China, Shanghai, Shanghai, China; Department of Infectious Diseases, Huashan Hospital, Fudan University, Shanghai, 200040, China, Shanghai, Shanghai, China; Institute of Antibiotics, Huashan Hospital, Fudan University, Shanghai, 200040, China; Key Laboratory of Clinical Pharmacology of Antibiotics, National Health and Family Planning Commission, Shanghai 200040, China, Shanghai, Shanghai, China

## Abstract

**Background:**

Nirmatrelvir/ritonavir (Paxlovid) is one of the few therapeutic options for COVID-19. It is not recommended to use in patients with end-stage renal disease (ESDR), and large-scale clinical trials are needed to support its application in this population. We investigated the pharmacokinetics of nirmatrelvir/ritonavir (150 mg/100 mg twice a day) in COVID-19 patients with hemodialysis (HD).

**Methods:**

SARS-Cov-2 tested positive patients with HD were included and given treatment with nirmatrelvir/ritonavir (150 mg/100 mg twice a day) for 5 days. For most patients, a 4 h-HD started 2 h after the morning dose on day 2. Blood samples were collected 4 h and 12 h after the first dose on day 1 to obtain peak and trough concentrations, respectively. And the blood samples at the start and finish time points of HD were drawn to observe the effect of dialysis on drug clearance. A tandem mass spectrometry (LC-MS/MS) method was conducted to quantify the nirmatrelvir and ritonavir concentrations.

**Results:**

A total of 14 COVID-19 patients undergoing dialysis were enrolled, with 4 females and 10 males. The age was 66.6 ± 8.0 years and the body mass index (BMI) was 22.3 ± 3.5. The peak concentration of nirmatrelvir after the first dose was 4960 ± 1889 ng/mL. And the trough concentration was 2940 ± 1305 ng/mL, which was approximately 10 times above the EC_90_ (292 ng/mL). Nirmatrelvir concentration fell by 42% from 10426 ± 3191 ng/mL to 6035 ± 2402 ng/mL during 4 h-HD. The peak and trough concentrations of ritonavir were 816 ± 445 ng/mL and 326±201 ng/mL after the first dose, respectively. And the concentration fell by 17% during 4 h-HD. Based on the assumption of a first-order elimination kinetic, nirmatrelvir/ritonavir 150 mg/100 mg, once a day dose regimen was simulated which showed that the plasma concentration of nirmatrelvir could also achieve the target EC_90_ value.

**Figure 1.**
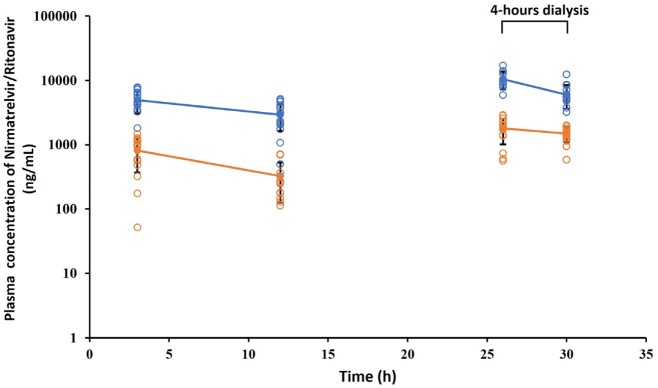
Plasma concentration of nirmatrelvir (blue circles) and ritonavir (orange circles) in COVID-19 patients with hemodialysis during the course of 150 mg/100 mg twice a day treatment. 4 h- hemodialysis was performed at 26 - 30 h after the first dose. Data are presented as the mean ± standard deviation. The open circles indicate the individual concentration data.

**Figure 2.**
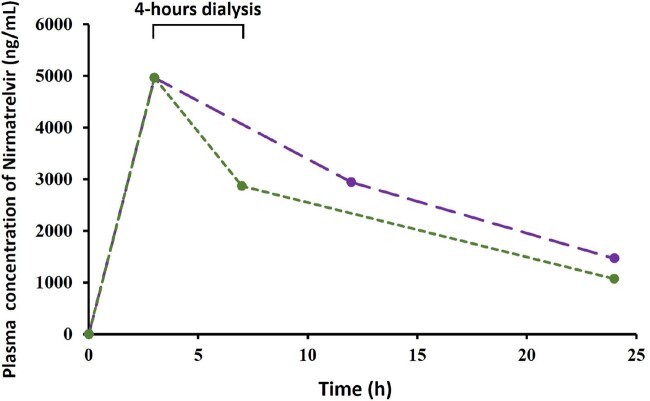
Simulated plasma concentration data in dialysis patients following a single dose of 150 mg/100 mg nirmatrelvir/ritonavir. Long dashed line represents patients not on dialysis. Short dashed line represents patients receiving a 4 h - dialysis.

**Conclusion:**

150 mg/100 mg twice a day dose regimen of nirmatrelvir/ritonavir produced sufficient concentrations to reach the effective target in COVID-19 patients with HD. Simulation data suggested that a modified dose with 150 mg/100 mg once a day could also reach effective concentration. Our data provide evidence for the application of nirmatrelvir/ritonavir in patients with ESRD and further validation in large-scale investigation was needed.

**Disclosures:**

**All Authors**: No reported disclosures

